# Clearance of HBeAg and HBsAg of HBV in mice model by a recombinant HBV vaccine combined with GM-CSF and IFN-α as an effective therapeutic vaccine adjuvant

**DOI:** 10.18632/oncotarget.25789

**Published:** 2018-07-13

**Authors:** Weidong Zhao, Gan Zhao, Shuren Zhang, Xianzheng Wang, Xueping Yu, Bin Wang

**Affiliations:** ^1^ Key Laboratory of Medical Molecular Virology of The Ministry of Health and Ministry of Education, School of Basic Medical Sciences, Fudan University, Shanghai, China; ^2^ Department of Infectious Diseases, Huashan Hospital, Fudan University, Shanghai, China

**Keywords:** GM-CSF, IFN-α, therapeutic vaccine, HBV, Ly6C^hi^ monocyte

## Abstract

Chronic hepatitis B virus (CHB) infection is a significant public threat. Current interferon-α (IFN-α) based therapies and anti-viral drugs have failed to clear the infection in the majority of CHB patients and animal models. In our previous study, we established a combined protocol that employed a 3-day pretreatment with granulocyte-macrophage colony stimulating factor (GM-CSF) prior to a standard HBV vaccine. It achieved a 90% reduction of HBsAg level in the HBsAg transgenic mouse model. This protocol, while effective, remains too complex for clinical use. In this study, we formulated a new regimen by combining GM-CSF, IFN-α and a recombinant HBV vaccine (GM-CSF/IFN-α/VACCINE) into a single preparation and tested its efficacy in a HBV infection model. After four vaccinations, both serum HBeAg and HBsAg were cleared, accompanied by a 95% reduction of HBV^+^ hepatocytes and the presence of a large number of infiltrating CD8^+^ T cells in the liver. Mechanistically these robust responses were initiated by a vaccine-induced conversion of CCR2-dependent CD11b^+^Ly6C^hi^ monocytes into CD11b^+^CD11c^+^ DCs. This finding sheds light on the potential mechanism of action of the GM-CSF-based vaccine adjuvant and provides definable markers for clinical assessment during future testing of such highly potent vaccine protocols in HBV patients.

## INTRODUCTION

Hepatitis B virus (HBV) infection remains as a major global cause of chronic hepatitis B (CHB), liver cirrhosis and hepatocellular carcinoma (HCC) [1]. In HBV-endemic areas, CHB is a leading risk of HCC [2]. The HBV genome encodes two main antigens, hepatitis B e antigen (HBeAg) and hepatitis B surface antigen (HBsAg). Pioneer studies have provided overwhelming evidence that the risk of HCC development in HBeAg-positive patients is significantly higher than in HBeAg-negative ones [3, 4]. Furthermore, presence of hepatitis B surface antigen (HBsAg) not only leads to immune-tolerance [5, 6], but also to a higher incidence of hepatocellular carcinoma [7, 8]. Therefore, seroconversion that is detected in HBeAg and HBsAg tests has become a desired clinical goal and often correlated with preventing HBV-related HCC [9, 10].

Immunological tolerance underlies viral persistence during chronic HBV infection [11, 12]. By the same token, the effectiveness of immunotherapeutic vaccines is linked to vaccine ability to break the tolerance. There are several HBV therapeutic vaccine approaches, based on DNA, peptide and dendritic cells as well as vaccines made of adjuvanted recombinant proteins. They are mainly assessed for potential utility by their associated disturbance of immunotolerance and the degree of T cell-mediated responses that could lead to clearance of infected cells [13–16]. Yet, none are in routine clinical use due to their various deficiencies. For instance, HBsAg protein vaccines gave minimal induction of anti-pre-S2/S antibody in chronic HBV patients [17]. DNA vaccinations that encoded small and middle envelope proteins activated transient and weak T-cell responses and in only a minority of the treated HBV carriers [18]. Yoon and colleagues demonstrated that HB-110, a DNA therapeutic vaccine for HBV treatment, exhibited weaker ability of mediating HBV-specific T cell immunity and HBeAg seroconversion in Korean than in Caucasian patients and animal models [19]. The data from a multicenter clinical trial showed that overstimulation with HBsAg-HBIG immunogenic complex therapeutic vaccine lead to immune fatigue and actually decreased the host immune response [20]. Another HBV therapeutic vaccine, GS-4774, which was formulated as a heat-inactivated yeast-based vaccine, was designed to evoke immune response against HBV in CHB patients. However, GS-4774 recently failed to meet its primary endpoint in a randomized phase II study [21]. Therefore, the search for an ideal HBV vaccine candidate is an ongoing effort and using cytokines to adjuvant vaccines is an option.

GM-CSF plays an essential role in the proliferation, differentiation, and survival of myeloid lineage cells [22]. Due to its pleiotropic effects on different cell lineages, GM-CSF has been used as an adjuvant to elicit immune responses for vaccine development against several infectious diseases and tumors [23, 24]. Essentially all of the attempts have had limited success. One potential reason for the failure is that, under some circumstances, the use of GM-CSF as a vaccine adjuvant can induce suppressive immune responses [25, 26], e.g. by induction of immature DC [27]. Interestingly, we found that such a negative regulation could be tweaked to become immune-activating simply by altering the timing of delivery. The undesirable negative impact of GM-CSF as an adjuvant was solved by using our recently published protocol in which 3-day pretreatments with GM-CSF were given before a vaccination into the same site of animals to elicit potent immune responses. This protocol overcame the immune tolerance and over 90% reduction of HBsAg level was observed in the HBsAg transgenic mouse model [28]. While the outcome remains exciting, the protocol by its original design was too complex for potential use in humans as a therapeutic vaccine.

IFN-α, the first substance licensed for CHB therapy, can accelerate DC maturation and promote co-stimulation factor expression and pro-inflammatory cytokine secretion in the presence of GM-CSF [29–31]. In particular, evidence is accumulating that GM-CSF and IFN-α can act together as an effective vaccine adjuvant for antitumor and antiviral immunity in both mouse models and human diseases [32, 33]. A prospective randomized trial in CHB treatment showed that the combination of GM-CSF and IFN-α was effective in non-responders to IFN-α monotherapy [34]. However, the use of GM-CSF plus IFN-α as a therapeutic HBV vaccine adjuvant is still not well defined.

In this context, the optimal cocktail therapy that consisted of IFN-α, GM-CSF, and recombinant HBV vaccine (VACCINE) was tested in a mouse model using recombinant adeno-associated virus 8 (AAV8)-1.3HBV infection. The aim of this study was to assess the immunological efficiency of the combined therapy and explore the role that the GM-CSF plus IFN-α adjuvant played in this therapeutic HBV vaccine protocol.

## RESULTS

### GM-CSF plus IFN-α combined with HBV vaccine induces robust immune responses

DTH is a well-established assay for antigen specific cellular responses to vaccination. To identify the most robust combination formula to induce cell-mediated responses, the HBV vaccine was tested with GM-CSF plus IFN-α as adjuvant at various dose formulations in wild-type C57BL/6 male mice. By using HBsAg as a re-challenging antigen on day 7 after the second immunization, we found that 10 μg of GM-CSF and 10,000 IU of IFN-α combined with 1 μg HBV vaccine (GM-CSF/IFN-α/VACCINE hereafter) could augment DTH responses to HBsAg more strongly than other formulations (Supplementary Figure 1A). Similarly, the anti-HBsAg antibody level was consistently and significantly increased the most with this formulation as compared with other combinations (Supplementary Figure 1B). In fact, excess of GM-CSF was associated with reduced responses. This optimal combination was therefore selected as the base preparation for further analyses.

### GM-CSF/IFN-α/VACCINE induces HBsAg sero-clearance and enhances humoral response in an infected mouse model

A mouse model for persistent HBV infection was established by employing the AAV8-1.3HBV virus in which 1.3 copies of HBV genome are packaged in a liver-tropic type 8 AAV vector [35]. The AAV8-1.3HBV virus was found to continually release complete HBV virions and express HBeAg, HBsAg, and HBcAg for more than six months. Since HBV-induced immunotolerance was also found in this mouse model, the AAV8-1.3HBV mouse has been accepted as an animal model for chronic hepatitis B immunotherapy studies [35–37], where the general indicators of immunotolerance breaking in HBV studies are the loss of expression of HBeAg and HBsAg in parallel with the respective antiserum conversions.

**Figure 1:**
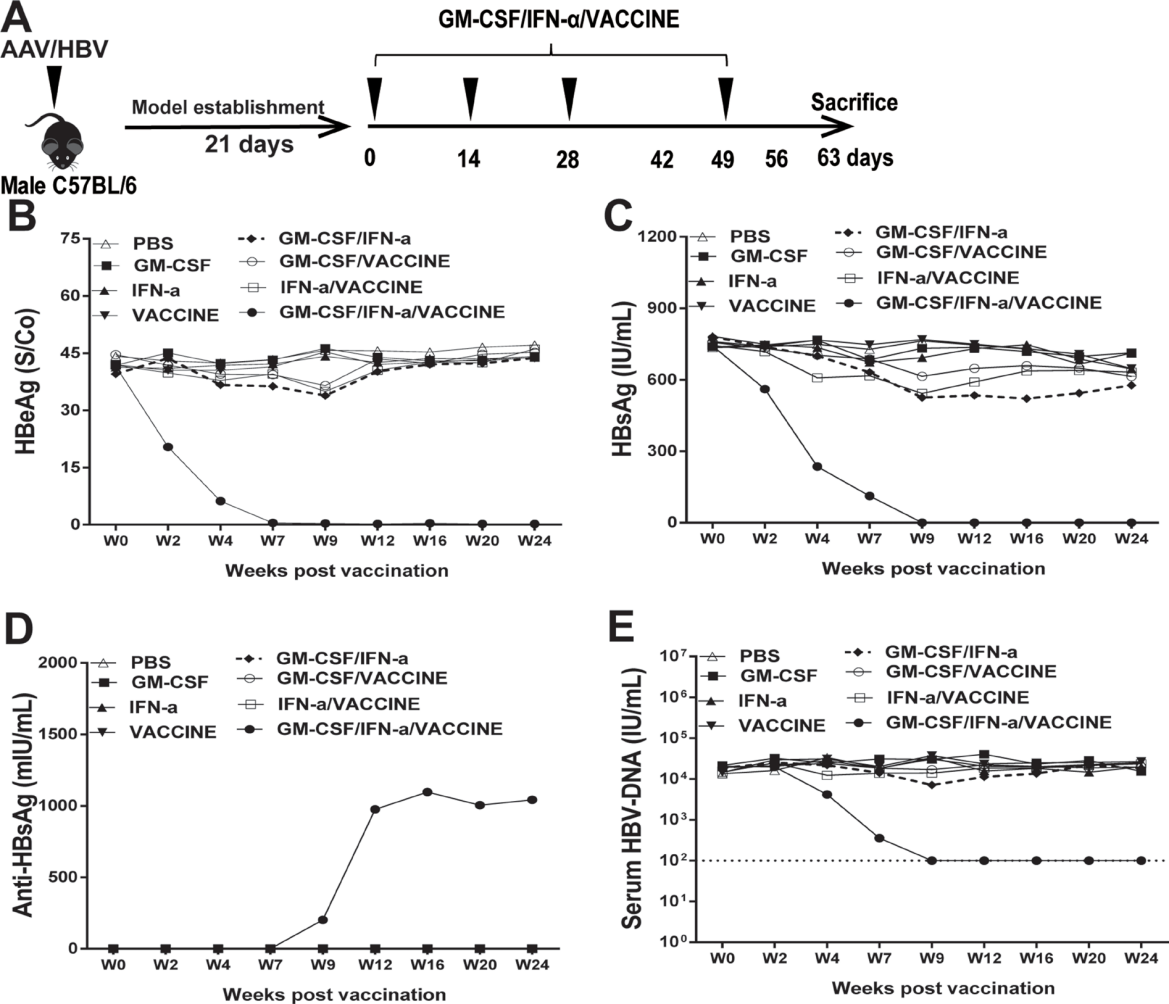
Treatment of AAV8-1.3HBV-infected mice with GM-CSF plus IFN-α combined with VACCINE (GM-CSF/IFN-α/VACCINE) decreased the infection (**A**) Schematic illustration of immunization workflow. Mice were immunized on day 21, 35, 49, and 70 post-infection. (**B**–**E**) At intervals up to 24 weeks, blood samples were collected at the indicated time points (week 0, 2, 4, 7, 9, 12, 16, 20, and 24) and the sera tested for HBV DNA, HBeAg and HBsAg, and HBsAb. The dotted line in E represents the assay limit of detection.

First, to evaluate whether the GM-CSF/IFN-α/VACCINE has the ability to reduce serum levels of both HBeAg and HBsAg, the immunotolerogenic animal model with AAV8-1.3HBV infection was established and then immunized with a mixed GM-CSF/IFN-α/VACCINE regimens intramuscularly for 3 times at biweekly intervals, and a boost injection three weeks after the third immunization (Figure 1A). Controls were treated with PBS, GM-CSF, IFN-α, or VACCINE alone or with GM-CSF/IFN-α, GM-CSF/VACCINE or IFN-α/VACCINE combinations. The sera of immunized AAV8-1.3HBV mice were collected for HBeAg, HBsAg, HBV DNA, anti-HBeAg antibody and anti-HBsAg antibody tests. The liver tissues were collected for HBV DNA, immunological histological chemistry (IHC) and pathology tests. The GM-CSF/IFN-α/VACCINE regimen induced anti-HBeAg antibody (Supplementary Figure 2A) and cleared the e antigens in serum (Figure 1B). Also, this regimen induced HBsAg-specific antibody (Figure 1D) and cleared HBsAg antigens in serum (Figure 1C). These are in drastic contrast to the other treatments, including treatment with vaccine alone. The clearance of HBeAg and HBsAg were sustainable for at least 24 weeks after the last immunization without rebounds in the GM-CSF/IFN-α/VACCINE group, whereas no significant changes were observed in the levels of HBeAg and HBsAg in the controls. Only the mixed GM-CSF/IFN-/VACCINE group induced a significant HBsAb response (Figure 1E). Most importantly, the serum HBV DNA was also almost undetectable at the end of the study (Figure 1B).

**Figure 2:**
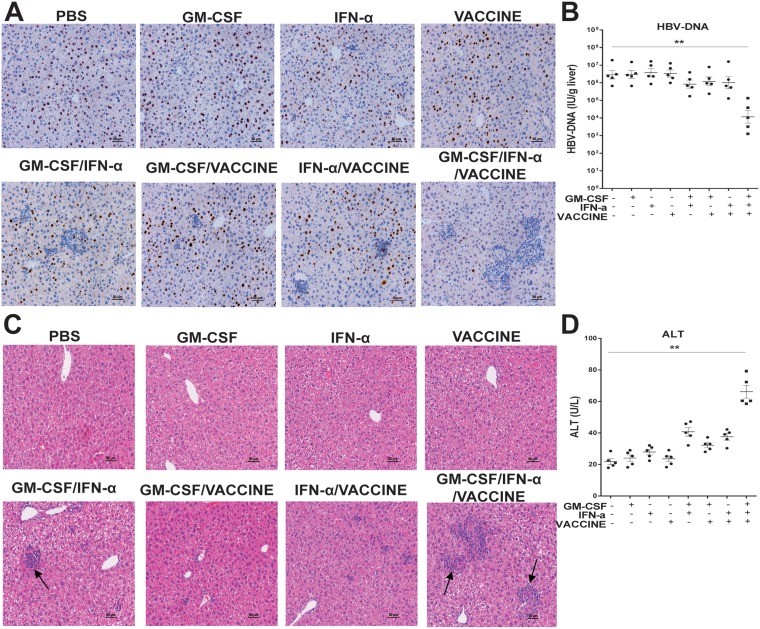
The GM-CSF/IFN-α/VACCINE promoted the clearance of HBcAg-positive hepatocytes (**A**) 14 days after the fourth immunization, liver sections were stained for HBcAg (brown staining) by IHC. Scale bar represents 50 μm. (**B**) 14 days after the fourth immunization, the HBV DNA levels in liver were analyzed by q-PCR. (**C**–**D**) 14 days after the fourth immunization, serum ALT was measured and the liver-infiltrating lymphocytes were stained by H&E. Scale bar represents 50 μm. Symbols represent mean ± SEM. ^*^P < 0.05; ^**^P < 0.01.

Since removal of viral DNA, particularly cccDNA of HBV, has been considered a cure, we assessed if our strategy could eliminate the DNA from the liver. Although the animal model does not generate cccDNA as it does in human, HBV genomic DNA can be retained in hepatocytes [37]. We first examined if HBcAg^+^ hepatocytes were cleared by the GM-CSF/IFN-α/VACCINE immunizations and observed that a significant amount of the HBcAg^+^ hepatocytes were eliminated (Figure 2A). Furthermore, the liver HBV DNA levels had declined > 2 log_10_IU/g in the GM-CSF/IFN-α/VACCINE group, as compared to the controls (Figure 2B). In addition, there was strong association with lymphocyte infiltration found in the GM-CSF/IFN-α/VACCINE-treated mouse livers (Figure 2C). Next we sought to assess any cytolytic effect on the liver after these treatments. Levels of serum ALT were monitored on weeks 0, 2, 4, 7, 9, 12 and 16 after commencing the immunizations. In the GM-CSF/IFN-α/VACCINE group a transiently elevating serum level of ALT was observed, which fell back to the normal level five weeks after the last vaccination. Similar but less extended transient elevation of ALT was also found in the other groups, excepting the PBS control (Figure 2D and Supplementary Figure 2). Interestingly, the elevated levels of ALT in the GM-CSF/IFN-α group approached that seen in GM-CSF/IFN-α/VACCINE group. Yet, only the GM-CSF/IFN-α/VACCINE group cleared HBsAg and HBeAg; the GM-CSF/IFN-α group did not (Figure 1F and 1G). It seems that the GM-CSF/IFN-α regimen perhaps induced only non-specific immune responses whereas the GM-CSF/IFN-α/VACCINE regimen additionally elicited the HBV-specific immune responses. Collectively, these results demonstrated that the GM-CSF/IFN-α/VACCINE can break established immune-tolerance in the HBV infection model, thereby eliciting a spectrum of robust anti-HBV responses that can result in a significant clearance of the viral infection.

### Induction of robust cell-mediated response *in vivo*

**Figure 3:**
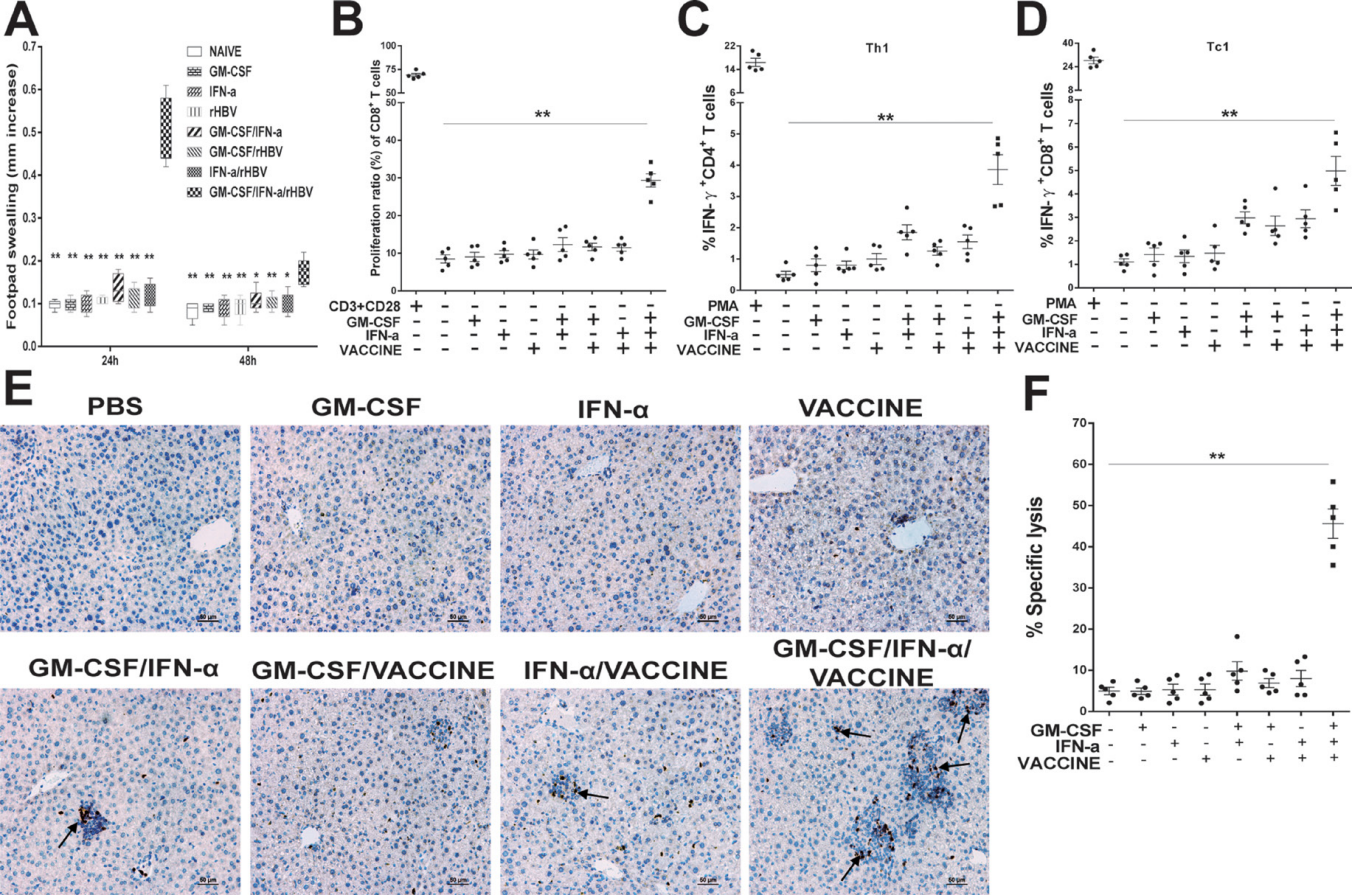
The GM-CSF/IFN-α/VACCINE elicited cellular immunity against AAV8-1.3HBV infection (**A**) 14 days after the fourth immunization of the infected mice, a total of 10 μg HBsAg was injected into the left footpad and PBS was injected into the right footpad as a control. Changes in footpad thickness were measured 24 and 48 h after injection to analyze DTH reaction. (**B**) 14 days after the fourth immunization, CFSE was added at a concentration of 1 μM to washed splenocytes (5 × 10^5^ cells). The cells were incubated for 10 min at 37°C in the dark, and labeling was stopped by adding RPMI 1640 supplemented with 10% fetal bovine serum (FBS). The cells were washed 3 times with medium and transferred to 96-well plates. Then the cells were incubated with HBsAg-specific CTL epitope S_208-215_ (ILSPFLPL; H-2^b^-restricted) as a specific stimulator in the presence of anti-CD28 (100 ng/mL). Cells incubated with anti-CD3 (1 μg/mL) and anti-CD28 (100 ng/mL) were used as positive controls. The plates were incubated at 37°C in a humidified atmosphere with 5% CO_2_ for 72 h before the labeled cells were determined by flow cytometric analysis. The proliferation ratio of CD8^+^ T cells is presented. C., D. 14 days after the fourth immunization, the splenocytes were stimulated with rHBsAg (10 μg/mL) for 18 h. All samples included addition of 10 μg/ml BFA for the last 6 h of incubation. PMA/ionomycin (100 ng/mL/1 μg/mL) was used as a positive control. Th1 (**C**) and Th2 (**D**) cells were then detected by intracellular cytokine. (**E**) IHC for CD8^+^ T cells (brown staining) on liver sections at 14 days after the fourth immunization. Scale bar represents 50 μm. (**F**) Characterization of HBsAg-specific CTL activity *in vivo* in AAV8-1.3HBV infected mice. Percentage of HBsAg-specific CTL activity *in vivo* is summarized. Bars are shown as mean ± SEM. ^*^P < 0.05; ^**^P < 0.01.

To clear infected hepatocytes, robust antigen specific cellular response is essential. DTH responses in AAV8-1.3HBV-infected mice immunized with different formulations were compared. After using HBsAg at 10 μg/foot-pad as a re-challenging antigen at 14 days after the fourth immunization, the result was that the animals in the GM-CSF/IFN-α/VACCINE treated group produced the highest level (*P* < 0.01) of footpad swelling 24 h after the sensitization (Figure 3A). To examine the proliferative ability of HBsAg-specific CD8^+^ T cells after vaccination, splenic cells labeled with CFSE were incubated *in vitro* with HBsAg-specific CTL epitope S_208-215_ (ILSPFLPL; H-2b-restricted, 1 μg/L) as a specific stimulator for 72 h in the presence of anti-CD28 (100 ng/mL). As shown in Figure 3B, the proliferative responses of HBsAg-specific CD8^+^ T cells were significantly enhanced in the GM-CSF/IFN-α/VACCINE-treated group compared with the other groups.

To gain mechanistic insight into this potent formulation, we analyzed several parameters of cellular immune responses. When splenocytes from the persistently infected mice that had been treated with GM-CSF/IFN-α/VACCINE were re-stimulated *in vitro* with 10 μg/mL HBsAg, a markedly higher frequencies of IFN-γ and IL-4 secreting CD4^+^ T cells were found, much greater than in other groups (Figure 3C and Supplementary Figure 3A). Classical cytotoxic T cells are key to clearing virally infected cells. The GM-CSF/IFN-α/VACCINE regimen induced significantly more HBsAg-specific IFN-γ^+^CD8^+^ T cells compared to the other immunized groups, not only in the periphery (Figure 3D and Supplementary Figure 3B), but also in the liver, where a large number of CD8^+^ T cell infiltrates was seen by IHC (Figure 3E). To directly assess *in vivo* the killing function of the activated CD8^+^ T cells elicited by the GM-CSF/IFN-α/VACCINE, splenocytes from naïve donor C57BL/6 mice were divided into two parts. One was labeled with 15 μM of CFSE and pulsed with 1 μg/mL of HBsAg-derived CTL peptide S_208-215_ (defined as CFSE^high^ target cells). The other one was labeled with 1 μM of CFSE and pulsed with 1 μg/mL of OVA-derived CTL peptide OVA_257-264_ (defined as CFSE^low^ target cells and providing a non-HBV target control). A mixture of CFSE^high^ and CFSE^low^ cells at a 1:1 ratio was adoptively transferred intravenously into immunized recipients at 2×10^7^ cells per mouse on the 14^th^ day after the fourth vaccination. Eight hours later, splenocytes were isolated from the recipients and CFSE fluorescence intensities were analyzed. HBsAg-specific killing was almost 45% in the GM-CSF/IFN-α/VACCINE group and was < 10% in other groups (Figure 3F and Supplementary Figure 3C). Taken together, these data indicate that the GM-CSF/IFN-α/VACCINE regimen can evoke robust HBsAg-specific cell-mediated immune responses in the immune-tolerogenic model created by AAV8-1.3HBV infection. As no specific TH polarization was found, this new regimen appears to enhance the overall cellular immunity, rather than targeting a specific sub arm of the adaptive immunity.

### Activation of CD11b^+^CD11c^+^DC

**Figure 4:**
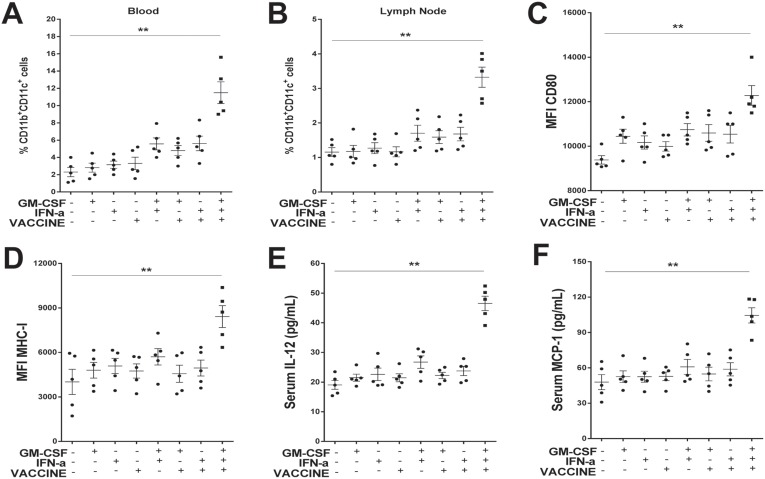
GM-CSF/IFN-α/VACCINE promoted both the production and the function of CD11b^+^CD11c^+^ DC One day after the second immunization, blood and inguinal lymph node samples were collected and analyzed. (**A**–**B**) Percentage of CD11b^+^CD11c^+^ DC in blood and in inguinal lymph nodes of AAV8-1.3HBV-infected mice inoculated with different formulations of the vaccine. (**C**–**D**) Comparison of markers on of CD11b^+^CD11c^+^ DC in blood: CD80 and MHC-I. (**E**–**F**) Serum concentration of IL-12 and monocyte chemoattractant protein-1 (MCP-1). Bars represent mean ± SEM. ^**^P < 0.01.

Effective activation of antigen presenting cells (APC) is the key to mounting effective innate and adaptive immune responses against invading pathogens [38]. By design, the primary function of adjuvants is to activate these cells, particularly DCs. After responding to the GM-CSF/IFN-α/VACCINE in the AAV8-1.3HBV model for 24 h, APC in PBMC and local lymph nodes were consecutively collected and examined. The percentage of CD11b^+^CD11c^+^ DC in the GM-CSF/IFN-α/VACCINE group was on average 5-fold higher in the blood (Figure 4A and Supplementary Figure 4A) and 3-fold higher in local lymph nodes (Figure 4B) compared to the control groups. In contrast, plasmacytoid DC (CD11b^-^ PDCA-1^+^, Supplementary Figure 4B) and CD11b^+^F4/80^+^macrophages (Supplementary Figure 4C) were not significantly altered. To determine the properties of this newly activated CD11b^+^CD11c^+^ DC population, we examined changes in the expression of functional markers, CD80, MHC-I and MHC-II. In the GM-CSF/IFN-α/VACCINE group, the expression of CD80 (Figure 4C), MHC-I (Figure 4D) and MHC-II (Supplementary Figure 4D) were significantly increased as compared with the control groups. Since cytokines play vital roles in modulating immune responses, we evaluated whether the GM-CSF/IFN-α/VACCINE could synergistically affect cytokine production. Some key inflammatory cytokines and chemokines in serum were determined by ELISA. Increased production of serum IL-12 (Figure 4E) and IL-4 (Supplementary Figure 4E) was observed (*P* < 0.01). A similar trend (*P* < 0.01) was found for monocyte chemo-attractant protein-1 (MCP-1), a chemokine that attracts monocytes infiltrating into the inflammation site (Figure 4F). Hence, the results revealed that the GM-CSF/IFN-α/VACCINE-mediated immune enhancements can be at least partially explained by a heightened CD11b^+^CD11c^+^ DC activation *in vivo*.

### GM-CSF/IFN-α/VACCINE drives the production of CD11b^+^Ly6C^hi^ monocytes

**Figure 5:**
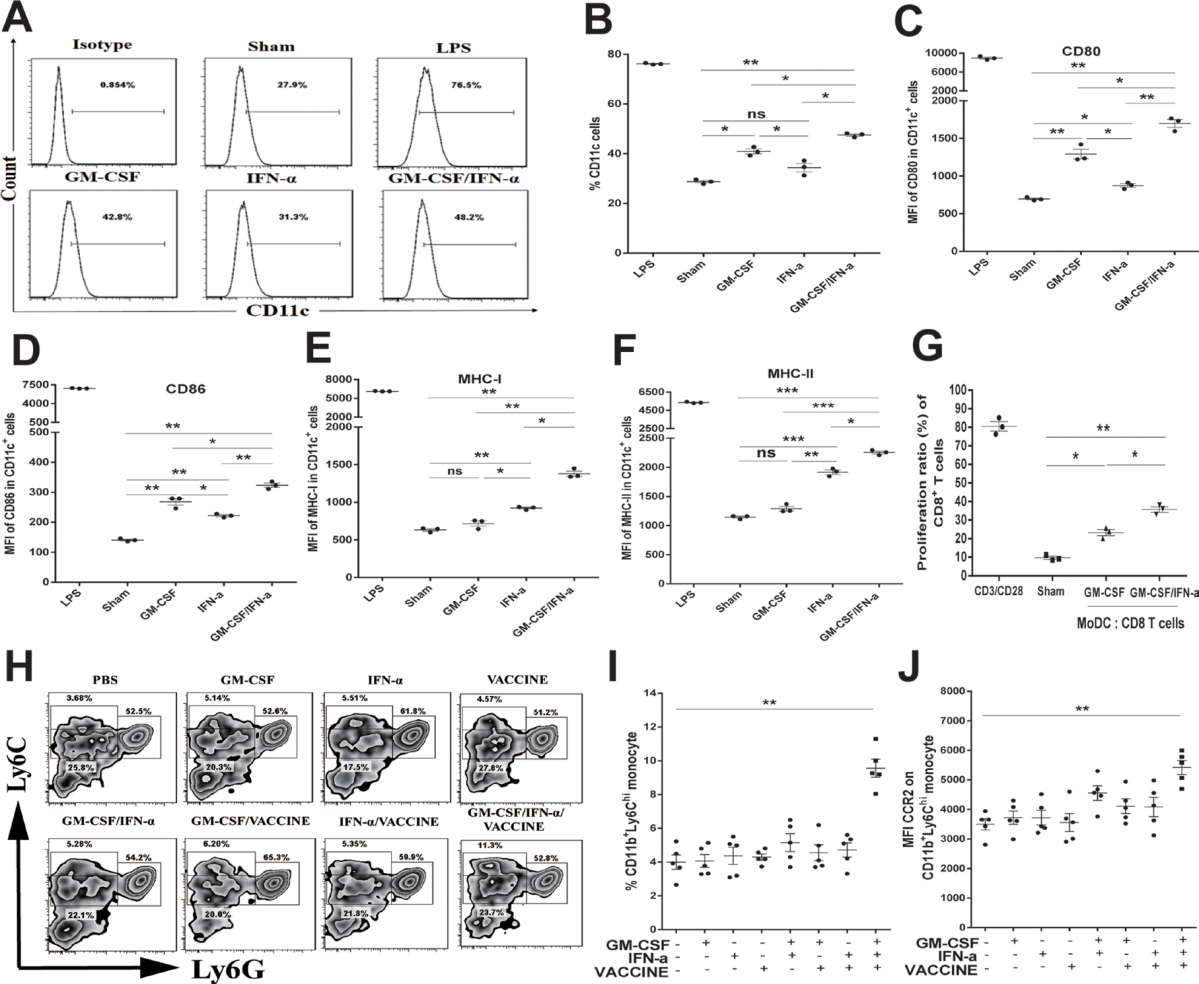
GM-CSF plus IFN-α promoted both the production and the function of CD11c^+^ monocyte derived DC (MoDC) *in vitro* and *in vivo* (**A**) CD11b^+^Ly6G^–^ monocytes were sorted from PBMC of male wild-type C57BL/6 mice (*n* = 50), then treated with mGM-CSF (50 ng/mL) and mIFN-α (50 IU/mL) once per day for 3 days before the frequencies of CD11c^+^ cells (MoDC) were determined. LPS (1 μg/mL) was used as a positive control. (**B**) The percentage of CD11c^+^ DC induced by different cytokine formulations is shown. (**C**–**F**) Comparison of CD80, CD86, MHC-I, and MHC-II on the variously induced CD11c^+^ MoDC. (**G**) MoDC were further cultured for 72 h with splenic CD8^+^T cells from AAV8-1.3HBV mice at a DC: T ratio of 1:10 and with HBsAg (10 μg/mL) then proliferative responses were assessed. Cells incubated with anti-CD3 (1 μg/mL) and anti-CD28 (100 ng/mL) were used as positive controls. The GM-CSF/IFN-α-induced MoDC promoted a significantly higher level of CD8^+^ T-cell proliferation than was promoted by GM-CSF-induced MoDC. (**H**–**J**) CD11b^+^Ly6C^hi^ monocytes and CD11b^+^Ly6C^lo^ monocytes in blood from AAV8-1.3HBV-infected mice that was collected 24 h after the second immunization with 3×GM-CSF+VACCINE were quantified by flow cytometry and expressed as a percentage of total CD11b^+^ cells (**H, I**). Numbers adjacent to outlined area indicate percent CD11b^+^Ly6C^hi^ monocytes (top left), CD11b^+^Ly6C^lo^ monocytes (bottom left), and CD11b^+^Ly6G^+^ granulocytes (right). The CD11b^+^ Ly6C^hi^ monocytes within the top left square were gated CCR2^+^. (**J**) Comparison of MFI of CCR2 on CD11b^+^Ly6C^hi^ monocytes of AAV8-1.3HBV-infected mice that had been immunized twice with different formulations of the vaccine. The cells were collected 24 h after the second immunization. Data are pooled from three independent experiments with 5 mice per group. Bars are shown as mean ± SEM. ^*^P < 0.05; ^**^P < 0.01; ^***^P < 0.001; ns, not significant.

Monocyte-derived DCs (MoDC) have been shown to be continuously generated from blood Ly6C^hi^ monocytes [39, 40]. To examine the effects of GM-CSF plus IFN-α on monocyte differentiation into MoDC, CD11b^+^Ly6G^-^ monocytes of wild type C57BL/6 mice were sorted from the peripheral blood (Supplementary Figure 5A) and treated with GM-CSF plus IFN-α for 3 days *in vitro*. Cells treated with LPS, GM-CSF or IFN-α alone or untreated were used as controls. Indeed GM-CSF plus IFN-α promoted a higher rate of conversion to CD11c^+^ DC than either GM-CSF or IFN-α used alone (Figure 5A and 5B). This was accompanied by a higher level of the co-stimulatory molecule CD80 (Figure 5C), CD86 (Figure 5D), MHC-I (Figure 5E) and MHC-II (Figure 5F), indicating a change similar to the *in vivo* observation. In a functional assay, GM-CSF/IFN-α-derived MoDC were primed with 10 μg/mL HBsAg for 24 h and then mixed with purified splenic CD8^+^ T cells (2 × 10^5^ cells/well) from AAV8-1.3HBV infected mice at a T:MoDC ratio of 10:1. A significantly higher level of CD8^+^ T cell proliferation was induced by incubation with the GM-CSF/IFN-α derived MoDC than was induced by other protocols (Figure 5G and Supplementary Figure 5B).

Having demonstrated that GM-CSF/IFN-α could effectively cause differentiation of monocytes into MoDC *in vitro*, it was important to study which subtypes of monocytes contributed to this process in the HBV immune-tolerant animals. We first infected mice with AAV8-1.3HBV and subsequently immunized with the GM-CSF/IFN-α/VACCINE twice with a 2-week interval, and then collected monocytes from PBMC 24 h later. An average 2.5-fold increase in the CD11b^+^Ly6C^hi^ monocytes was found in the blood 24 h later (Figure 5H and 5I). However, this treatment did not alter CD11b^+^Ly6C^lo^ monocyte or CD11b^+^Ly6G^+^ granulocyte numbers (Supplementary Figures 5C and 5D). CCR2 on Ly6C^hi^ monocytes plays an essential role in mediating their trafficking to inflammatory sites [41, 42] and an elevation of MCP-1 (a CCR2 chemotactic ligand) was found in the blood of GM-CSF/IFN-α/VACCINE-treated group (Figure 4F). Echoing this finding, CCR2 was significantly up-regulated among the CD11b^+^Ly6C^hi^ monocytes in the PBMC of the GM-CSF/IFN-α/VACCINE group, as compared with other groups (Figure 5J and Supplementary Figure 5E), suggesting that this receptor ligand interaction may be important for the migration of CD11b^+^Ly6C^hi^ monocytes to the infected sites of immunized animals.

### Blockage of Ly6C^hi^ monocytes abrogates HBV clearance in AAV8-1.3HBV model

**Figure 6:**
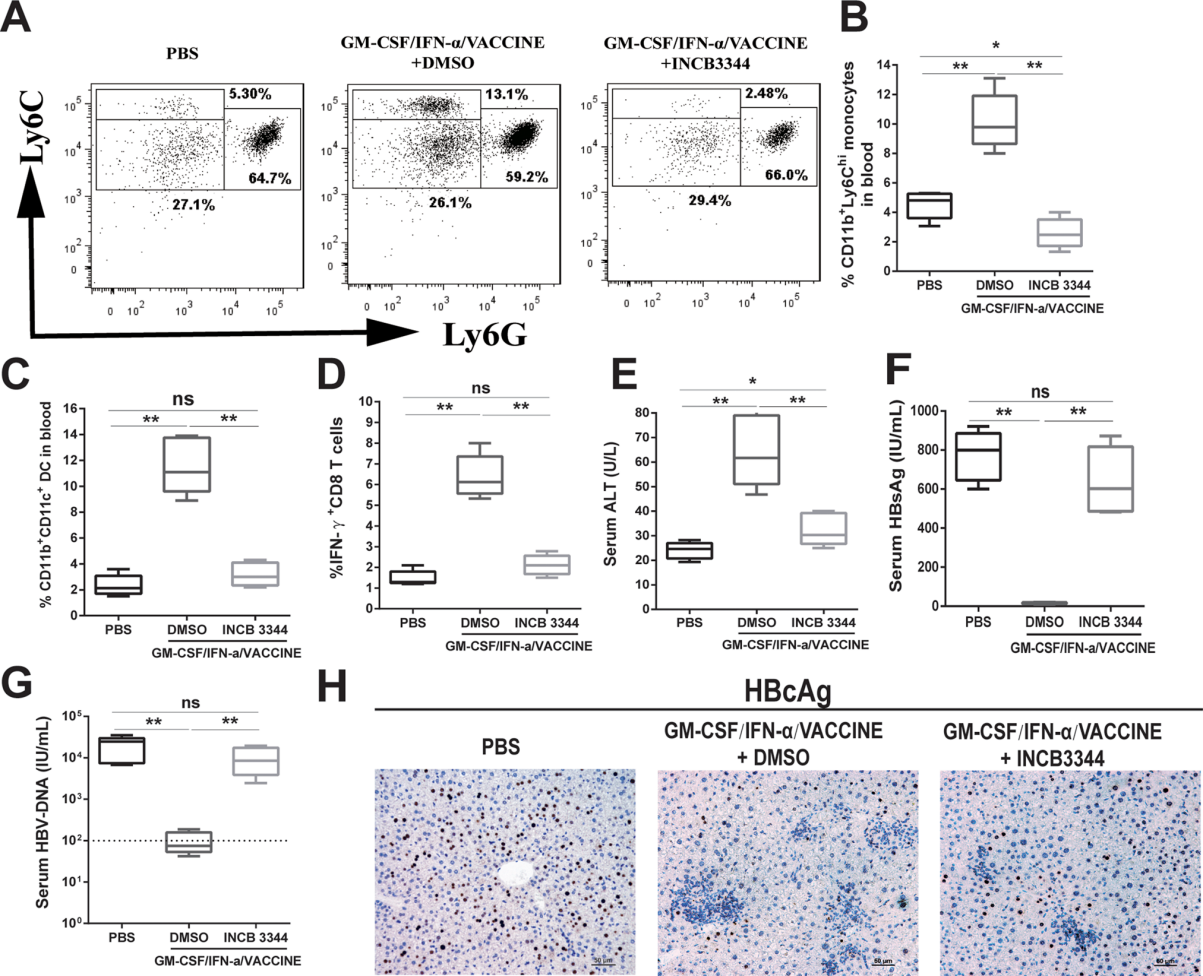
Blockage of Ly6C^hi^ monocytes abrogates the HBV clearance caused by GM-CSF/IFN-α/VACCINE treatment AAV8-1.3HBV infected mice were treated with CCR2 antagonist (INCB 3344, 30 mg/kg per day for 3 days) by intraperitoneal injection after the GM-CSF/IFN-α/VACCINE administration. The CD11b^+^Ly6C^hi^ monocytes was measured 12 h after the third INCB 3344 administration. (**A**) Representative flow-cytometric images are shown (*n* = 5–6 mice per group). (**B**) CD11b^+^Ly6C^hi^ monocytes were quantified by percentage of total CD11b^+^ cells. (**C**) CD11b^+^CD11c^+^ DC in blood were analyzed 12 h after the third INCB 3344 administration and quantified by percentage of total CD11b^+^ cells. (**D**–**G**) IFN-γ^+^CD8^+^ T cells in spleen, serum ALT, serum HBsAg, and serum HBV DNA were measured at 14 days after the fourth vaccination. The dotted line in G represents the assay limit of detection. Bars represent the mean ± SEM. ^*^P < 0.05; ^**^P < 0.01; ns, not significant. (**H**) 14 days after the fourth vaccination, the HBcAg in liver sections was stained by IHC (brown). Representative images are shown (*n* = 5 mice per group). Scale bar represents 50 μm.

We next determined if the conversion of Ly6C^hi^ monocytes to MoDC was essential to the robust adaptive immune responses and anti-viral effects associated with the combination regimen. INCB3344, a selective CCR2 antagonist [43–45] was injected i.p. at 30 mg/kg per day into AAV8-1.3HBV infected mice 1 h before the GM-CSF/IFN-α/VACCINE inoculation, and again on days 2 and 3. PBS and 10% DMSO were used as controls. PBMC were collected and analyzed 12 h after the third administration of INCB 3344. The selective blockade of CCR2 significantly decreased the generation of CD11b^+^Ly6C^hi^ monocytes (Figure 6A and 6B) and CD11b^+^ CD11c^+^DC (Figure 6C), without affecting CD11b^+^Ly6C^lo^ monocytes, CD11b^+^Ly6G^+^ granulocytes, T cells or NK cells (data not shown). This demonstrated that the GM-CSF/IFN-α/VACCINE-induced CD11b^+^CD11c^+^DC were derived from CD11b^+^Ly6C^hi^ monocytes in a CCR2-dependent manner.

To further define the importance of CCR2 expression on Ly6C^hi^ monocytes, we examined if CCR2 blockade could result in abrogation of the clearance of HBsAg. Fourteen days after the fourth immunization, serum samples were collected for ALT, HBsAg and HBV DNA detection by ELISA and splenocytes were harvested and re-stimulated with HBsAg (10 μg/mL) for 18 h *in vitro* to test their capacity for IFN-γ production. As depicted in Figures 6D and 6E, the use of INCB 3344 led to significant reduction of HBsAg specific IFN-γ-secreting CD8^+^ T cells as well as reduction of ALT level after the GM-CSF/IFN-α/VACCINE immunizations. Conversely, the beneficial effects of the combination vaccine was essentially lost in terms of the serum levels of HBsAg and HBV DNA or HBcAg-positive hepatic cells in infected mice (Figures 6F, 6G, and 6H). This result demonstrated that induction of Ly6C^hi^CCR2^+^ monocytes by the immunizations with GM-CSF/IFN-α/VACCINE was crucial to mounting effective adaptive anti-viral responses, particularly to markedly elevating the levels of antigen-specific IFN-γ-secreting CD8^+^ T effector cells.

## DISCUSSION

In this study, we observed that a regimen consisting of GM-CSF, IFN-α, and recombinant human HBV vaccine (VACCINE) administered into animals induced a robust antigen-specific immune response. This was characterized by 100% seroconversion of HBeAg and HBsAg along with a significant reduction of viral DNA in sera and elimination of HBcAg-positive hepatocytes in the AAV8-1.3HBV-infected mouse model. Further study revealed that the significant therapeutic effects were initiated by activation of Ly6C^hi CCR2+^ monocytes that are essential in recruitment and induction of CD11b^+^CD11c^+^ DC. The therapeutic effect of the GM-CSF/IFN-α/VACCINE administrations arose by breaking the HBV-induced immunotolerance. This resulted in the recruitment and conversion of monocytes into moDC and these generated the robust responses: HBsAg-specific T cell proliferation, cytolytic CD8^+^ T cell activity and B cell production of anti-HBsAg antibodies. This novel regimen thus provides a simple yet highly effective therapeutic treatment for CHB that has long been desired by the field.

GM-CSF was the first cytokine documented to promote differentiation of myeloid lineage cells into DC [46]. Due to its effects, GM-CSF has been exploited as an adjuvant for virus and cancer vaccines in different animal models [47, 48]. However, results in human trials were largely disappointing and sometimes associated with opposite effects [49, 50]. In contrast, some investigations had demonstrated that GM-CSF combined with other cytokines as a combined adjuvant was more effective than GM-CSF alone [51, 52]. This is again proven true in this and our previous work [28].

Monocytes are cells with high differentiation potential and can differentiate into M1 macrophages in the presence of GM-CSF and IFN-γ, or into DC on exposure to GM-CSF and IL-4 [53]. Since Ronald et al. first reported that monocytes could differentiate into DC that had potent ability to elicit T cell immunity on exposure to GM-CSF and IFN-α [54], multiple reports have recognized the benefit of IFN-α-MoDC vaccines [55, 56]. IFN-α is crucial for the generation of Ly6^hi^ monocytes [57] and can accelerate DC maturation, promote co-stimulation factor expression and pro-inflammatory cytokine secretion [29]. Since IFNα-MoDC has the capacity to cross-present antigen to CD8^+^ T cells, in contrast to IL-4-MoDC, there is growing interest in the use of IFNα-MoDC to treat tumors and infectious diseases [58–61]. Whether the DC that are generated from GM-CSF plus IFN-α in the current recombinant HBV vaccine actually facilitate the therapeutic treatment has not been established. This study is the first to demonstrate that the GM-CSF/IFN-α/HBV vaccine regimen could overcome immune tolerance and promote robust anti-HBV-specific immune responses. However, an optimal dose combination of HBV vaccine with GM-CSF and IFN-α preferentially promoted monocyte polarization into DC rather than macrophages, in line with DC having a superior role in immune activation. Here, the presence of viral antigen in the formulation was critical for the monocyte differentiation, suggesting an antigen-specific response was induced amongst the heightened inflammatory response.

HBV-specific CD8^+^ T cells are known to be the vital effectors contributing to the HBV clearance. Exhausted CD8^+^ T cells expressing inhibitory receptors, including PD-1, CTLA-4, and TIM-3 are thought to underlie the major defect in HBV viral clearance [62]. However, clinical trials with the anti-PD-1 against CHB have had only limited success [63], suggesting that a reactivation of these exhausted CD8^+^ T cells may not be sufficient; the intrahepatic virus-specific CTLs from chronic patients have higher PD-1 expression [64] and are less susceptible to functional restoration by PD-1/PD-L1 blockade [65], as compared to circulating virus-specific CTLs. An ideal approach therefore may be to freshly active the naïve CD8^+^ T cell pool. In our current study, the GM-CSF/IFN-α/VACCINE significantly promoted IFN-γ secretion by CD8^+^ T cells and enhanced a powerful CTL response mediating the elimination of HBcAg-positive hepatocytes. Although we demonstrated that the GM-CSF/IFN-α/VACCINE treatments could reduce the level of HBV DNA in serum, the liver HBV DNA was not completely cleared by the treatments. It appears that HBV-specific CTLs in the liver are insufficient to clear infected hepatocytes [66], suggesting that a subsequent treatment should be considered. Intrahepatic HBV-specific CD8^+^ T cells have been shown to be key to the functional recovery of anti-HBV immunity [67]. Consistent with this, we observed CD8^+^ T cell numbers increased in the livers in the GM-CSF/IFN-α/VACCINE group, suggesting recruitment from the peripheral blood. Moreover, we observed that IL-12, a key cytokine inducing naïve T cells differentiation into effector T cells was higher in the GM-CSF/IFN-α/VACCINE group than in other groups. These results suggest that the recruited CD8^+^ T cells contributed to the HBV clearance following the GM-CSF/IFN-α/VACCINE treatment.

Serum HBsAg levels have been found to reflect the transcriptional activity of the cccDNA; measurement can help predict disease progression in patients [68, 69], and studies have indicated that the levels of serum HBsAg contribute to the liver fibrosis severity in HBeAg-positive patients [70, 71]. In HBeAg-negative patients, serum HBsAg level < 1000 IU/mL can be used to define patients at lower risk of HCC [72]. Furthermore, Chen and colleagues demonstrated that 9-year cumulative HCC incidences were < 1% after the patients had anti-HBsAg and anti-HBeAg seroconversions [73]. In this study, HBeAg and HBsAg seroclearance occurred only in mice given the GM-CSF/IFN-α/VACCINE treatments. Notably, enhanced HBsAb productions were also observed by the GM-CSF/IFN-α/VACCINE treatments. Taking the evidence together, it may be that the progression of liver diseases could be delayed if CHB patients treated by the GM-CSF/IFN-α/VACCINE regimens.

Several limitations existed in this study. First, we observed that recombinant AAV/HBV infection could last for more than 10 months in C57BL/6 mouse and the AAV/HBV-infected mice did not respond to the routine recombinant HBs vaccine. These results demonstrated that recombinant AAV/HBV could chronically infect the mice and lead to immunotolerance in this mouse model. However, in the model the mice are infected with the recombinant AAV/HBV for 3 weeks at the time of the first vaccination, while human patients are chronically infected with HBV for decades. Hence there is a significantly different character of immune tolerance and exhaustion in this model compared to human patients. Further rigorous study is warranted. Second, how the hepatic immune populations responded to the GM-CSF/IFN-α/VACCINE treatments was not fully defined and future investigation is needed.

## MATERIALS AND METHODS

### Animals, virus and reagents

C57BL/6 male mice (six- to eight-week-old) were obtained from Huafukang Laboratory Animal Co. Ltd (Beijing, China). All mice were housed in individual specific pathogen-free ventilated cages following the Institutional Animal Care guidelines of Fudan University. AAV8-1.3HBV virus which carries 1.3 copies of HBV genome (genotype D, serotype ayw) packaged in AAV serotype 8 vector was purchased from FivePlus Molecular Medicine Institute (Beijing, China). Alum-absorbed Chinese hamster ovary cells-derived recombinant HBsAg (VACCINE, hereafter) and recombinant human GM-CSF were kindly gifted by the Jingtan Biotech Corp. of China North Pharmaceutical Group (Shijiazhang, China). Recombinant human Interferon-α (IFN-α) was purchased from Kawin Technology, Ltd (Beijing, China). Mouse GM-CSF and mouse IFN-α was bought from Sino Biological Inc (Beijing, China). Purified recombinant HBsAg (rHBsAg) was obtained from Guikang Biotechnology, Ltd (Shanghai, China).

### Animal model

Each mouse received 1 × 10^10^ TCID50 of AAV8-1.3HBV virus through a tail vein injection according to the manufacturer's instruction. Serum collected from infected mice was used to monitor HBV DNA, HBsAg, HBsAb and HBeAg for 14 days post-infection.

### Immunization

The AAV8-1.3HBV mice were randomly assigned to eight groups (*n* = 5) following the design shown in Table 1. GM-CSF (10 μg), IFN-α (10,000 IU), and VACCINE (1 μg) were injected subcutaneously following the immunization procedure on day 0. Three subcutaneous boosts were administrated on days 14, 28, 49.

**Table 1:** Immunization groups

Group	Dose
①PBS	-
②GM-CSF	10 μg
③IFN-α	10,000 IU
④VACCINE	1 μg
⑤GM-CSF/IFN-α	10 μg/10,000 IU
⑥GM-CSF/VACCINE	10 μg/1 μg
⑦IFN-α/VACCINE	10,000 IU/1 μg
⑧GM-CSF/IFN-α/VACCINE	10 μg/10,000 IU/1 μg

Forty AAV8-1.3HBV infected mice were randomly divided into eight experimental and control groups, with five mice in each group. GM-CSF (10 μg), IFN-α (10,000 IU), and VACCINE (1 μg) were injected subcutaneously following the immunization design. PBS was used as sham control.

### Generation of monocyte-derived DC (MoDC)

CD11b^+^Ly6G^-^ monocytes were sorted from PBMC of male wild-type C57BL/6 mice (*n* = 50) with Easysep™ mouse monocyte isolation kit (STEMCELL, Canada). CD11b^+^Ly6G^-^ monocytes were incubated at 37°C in a humidified 5% (v/v) CO_2_ air atmosphere in RPMI 1640 medium supplemented with 10% fetal bovine serum (v/v), penicillin (100 U/mL), and streptomycin (100 μg/mL). Monocytes were cultured in the medium with recombinant mouse GM-CSF (rmGM-CSF, 50 ng/mL), recombinant mouse IFN-α (rmIFN-α, 25 ng/mL, 2,000 IU/μg), and LPS (1 μg/mL) at 2 × 10^5^/mL in 24-well cell culture plates for 3 days. The cells in semi-suspension were identified as MoDC.

### *In vitro* MoDC and T cell co-culture

Conventional CD8^+^ T cells were sorted (MajoSort, Biolegend) from splenocytes of AAV8-1.3HBV infected mice and labeled with carboxyfluorescein diacetate succinimidyl ester (CFSE). GM-CSF derived or GM-CSF/IFN-α derived MoDC were incubated with HBsAg (10 μg/mL) for 24 h before being mixed with the purified CD8^+^ T cells (2×10^5^ cells/well) at a T:MoDC ratio of 10:1. After 3-day co-culture, cells were analyzed by flow cytometry.

### Delayed-type hypersensitivity (DTH) assay

On day 14 after the fourth vaccination, mice in each group were challenged with 10 μg of rHBsAg in the left footpad as a test and phosphate buffered saline in the right footpad as a control. Swelling was measured after 24 and 48 h using a micrometer. Results are presented as mean ± standard deviation in footpad thickness of left versus right footpad.

### *In vitro* T cell proliferation assay

On day 14 after the fourth immunization, all mice were sacrificed and single lymphocyte suspensions were prepared from spleens. A commercially available cell-proliferation dye (CFSE) was used to assess lymphocyte proliferation. This fluorochrome-tagged dye binds to cellular proteins containing primary amines and is distributed equally to daughter cells upon division; thus, as cells divide, fluorescent staining becomes less bright. *In vitro* T cell proliferation was measured as reported previously [74]. Briefly, CFSE was added at a concentration of 1 μM to washed splenocytes (5 × 10^5^ cells). The cells were incubated for 10 min at 37°C in the dark, and labeling was stopped by adding RPMI 1640 supplemented with 10% fetal bovine serum (FBS). The cells were washed 3 times with medium and transferred to 96-well plates. Then the cells were incubated with HBsAg-specific CTL epitope S_208-215_ (ILSPFLPL; H-2^b^-restricted, 1 μg/L) as a specific stimulator in the presence of anti-CD28 (100 ng/mL). Cells incubated with anti-CD3 (1 μg/mL) and anti-CD28 (100 ng/mL) were used as positive controls. The plates were incubated at 37°C in a humidified atmosphere with 5% CO_2_ for 72 h before the labeled cells were determined by flow cytometric analysis.

### Serological and biochemical analysis

Serum HBsAg, HBsAb and HBeAg were determined by ELISA kits purchased from Kehua Bio-engineering Co. Ltd (Shanghai, China). Serum IL-12, IL-4 and MCP-1 were tested by ELISA kits (eBioscience, USA). Serum alanine aminotransferase activity (ALT) was measured with ALT kit from BioSino Bio-technology and Science Inc. (Beijing, China).

### HBV DNA quantitation

Serum HBV DNA was determined by real-time quantitative PCR with a kit from Kehua Bio-engineering Co., Ltd (Shanghai, China). The detection lower limit was at 100 IU/mL.

### *In vivo* cytotoxic lysis assay

OVA-specific CTL epitope OVA_257-264_ (SIINFWKL) and HBsAg-specific CTL epitope S_208-215_ (ILSPFLPL; H-2^b^-restricted) were synthesized by Sangon Biotech (Shanghai, China). *In vivo* cytotoxic lysis assay was conducted as described previously [28]. Briefly, to provide non-HBV target controls, splenocytes from naïve C57BL/6 donor mice were labeled with 15 μM of CFSE and pulsed with 1 μg/mL of S208-215 (CFSE^high^ target cells). An equal fraction of splenocytes were labeled with 1 μM of CFSE and pulsed with 1 μg/mL of OVA257-264 (CFSE^low^ target cells). A mixture of CFSE^high^ and CFSE^low^ cells at a 1:1 ratio was adoptively transferred intravenously into immunized recipients at 2×10^7^ cells per mouse on the 14th day after the fourth vaccination. Eight hours later, the splenocytes were isolated from the recipients and CFSE fluorescence intensities were analyzed by FACS.

### Blockage of CCR2^+^ monocytes with INCB 3344

The AAV8-1.3HBV infected mice were randomly assigned to receive PBS, INCB 3344 (30 mg/kg per day for three days, *n* = 5, MedChem Express, USA), or vehicle (10% dimethylsulfoxide, DMSO, *n* = 5) via intra-peritoneal injection at 1 h before the GM-CSF/IFN-α/VACCINE inoculation. PBS, INCB 3344 or DMSO injection was repeated on days 2 and 3. Ly6C^hi^CCR2^+^ monocytes were tested by flow cytometry 24 h later.

### Histology and immunohistochemistry (IHC) analysis

For histological investigations livers were fixed with 4% formalin overnight and processed for paraffin embedding. 10-μm sections were stained with hematoxylin-eosin (H&E) and examined by light microscopy to determine liver lesions.

HBcAg-positive liver cells and infiltration inflammatory cells were further examined by IHC analysis. For staining of HBcAg and CD8, 10-μm sections of livers were fixed with ice-cold methanol for 30 min, dehydrated through graded alcohols and subjected to antigen retrieval using 10 mM sodium citrate. Sections were washed with tris borate saline Tween-20 (TBST) and then blocked with 2% bovine serum albumin (BSA) for 1 h. Liver sections were incubated with the respective mouse monoclonal anti-HBcAg or anti-CD8. Slides were then washed for 5 min in TBST and incubated for 1 h. After washing, section were incubated with goat anti-mouse horseradish peroxidase antibody for 1 h. Finally, slides were incubated with 3,3-diaminobenzidine (DAB) chromogen solution and then counterstained with hematoxylin. Slides were then observed under a Nikon microscope and NIS elements microscope software (Nikon). Ten different fields were examined in each case.

### Flow cytometry

Flow cytometry was performed on a FACS LSRFortessa flow cytometer with Diva software (BD Bioscience, USA). For DC analysis *in vitro* and *in vivo*, the cells were stained with CD11b (clone: M1/70, BioLegend), CD11c (clone: HL3, BD), CD80 (clone: 16-10A1, eBioscience), CD86 (clone: GL-1, BioLegend), MHCI (clone: AF6.88.5.5.3, eBioscience), MHCII (clone: M5/114.15.2, eBioscience), PDCA-1 (clone: eBio927, eBioscience). For monocytes analysis *in vivo*, the cells were stained with CD11b (clone: M1/70, BioLegend), Ly6G (clone: RB6-8C5, eBioscience), Ly6C (clone: HK1.4, BioLegend), CCR2 (clone: SA203G11, BioLegend). For intracellular cytokine detection, the splenocytes were stimulated with rHBsAg (10 μg/mL) for 18 h. All samples included addition of 10 μg/ml Brefeldin A (BFA, BD) for the last 6 h of incubation. Tests included: PMA/ionomycin (100 ng/mL/1 μg/mL) as positive control; Th1 (CD4 (clone: RM4-5, BioLegend); IFN-γ (clone: XMG1.2, BioLegend); Th2 (CD4 (clone: RM4-5, BioLegend); anti-IL4 (clone: 11B11, BioLegend)); Tc1 (CD8 (clone: 53-6.7, eBioscience); anti-IFN-γ (clone: XMG1.2, BioLegend)). Isotype-matched control mAb were used to identify non-specific background staining.

### Statistics

All results are presented as means ± Standard Error of Mean (SEM). For continuous variables, Mann-Whitney *U* test was used for abnormal distribution, the two-tailed Student's *t*-test for normal distribution and homogeneity of variance. A *P* value of < 0.05 was deemed significant for all analyses.

## SUPPLEMENTARY MATERIALS FIGURES


