# Supplementary material for: Clearance of HBeAg and HBsAg of HBV in mice model by a recombinant HBV vaccine combined with GM-CSF and IFN-α as an effective therapeutic vaccine adjuvant
Source: Oncotarget. 2018 Jul 13;9(76):34213–28. doi: 10.18632/oncotarget.25789 (PMC6188151; doi:10.18632/oncotarget.25789)
Supplement: Supplementary file 1 [file oncotarget-09-34213-s001.pdf]

Clearance of HBeAg and HBsAg of HBV in mice model by a recombinant HBV vaccine combined with GM-CSF and IFN-α as an effective therapeutic vaccine adjuvant

SUPPLEMENTARY MATERIALS

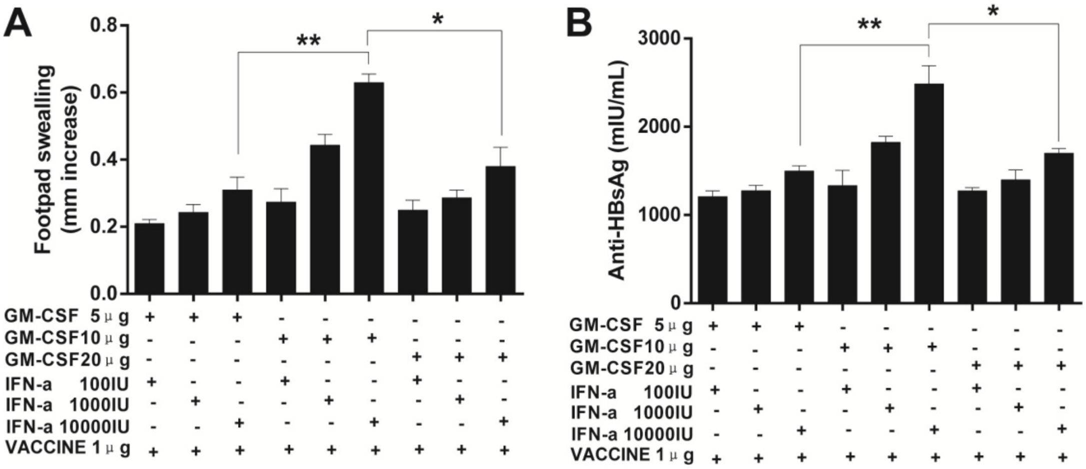

Supplementary Figure 1: GM-CSF combined with IFN-α enhanced immune response of HBV vaccine (VACCINE). The wild type C57BL/6 mice were immunized three times with VACCINE (1 μg) combined with different dose formulation of GM-CSF and IFN-α in a 2-week interval. (**A**) 7 days after the third immunization, DTH were tested at 24 hours after HBsAg challenge at footpads of immunized mice. (**B**) 7 days after the third immunization, serum of immunized mice were collected for anti-HBsAg test by ELISA. Bars represent mean ± SEM from 3 independent experiments, $^{*}P < 0.05$; $^{**}P < 0.01$; ns, not significant.

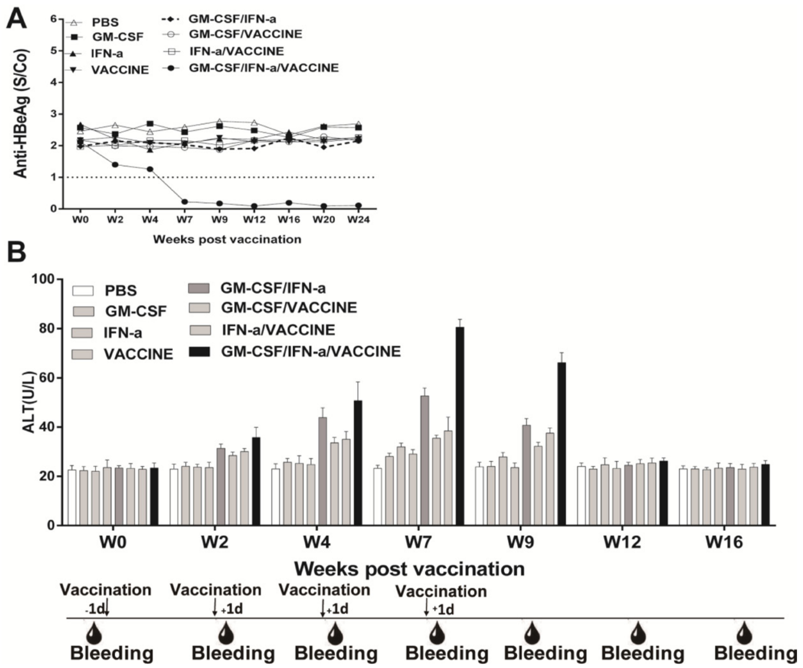

Supplementary Figure 2: Responses of AAV/HBV infected mice to the GM-CSF/IFN-α/VACCINE regimen. (**A**) At intervals up to 24 weeks, serum anti-HBeAg was determined by ELISA. Dotted lines represent the threshold of detection. >1 means anti-HBeAg-negative, and <1 means anti-HBeAg-positive. (**B**) One day before the first vaccination, the blood samples were collected from the AAV/HBV infected mice. Serum ALT was determined as a background level. Then the model mice were bled one day after the rest of three immunizations. After the end of vaccination, the mice were bled at 2-week, 3-week and 4-week follow up.

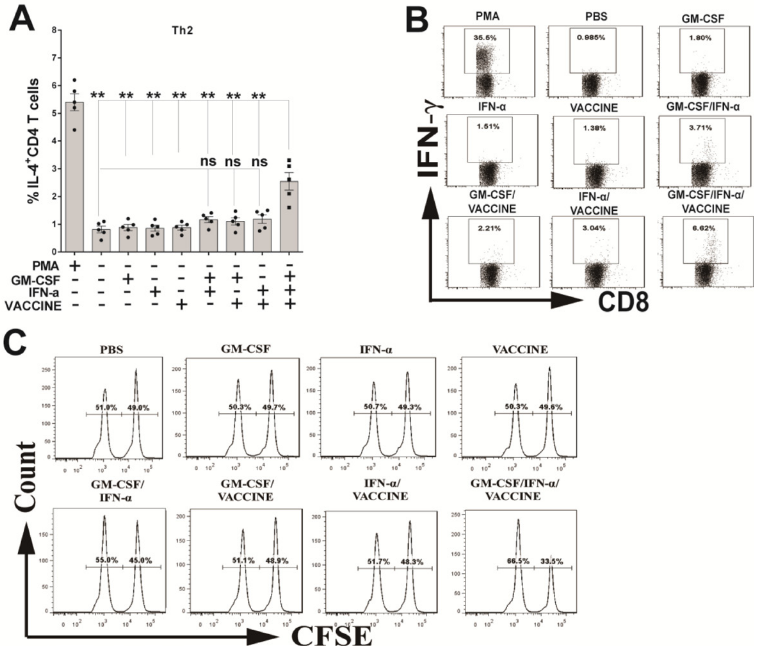

Supplementary Figure 3: GM-CSF/IFN-α/VACCINE elicit cellular immunity against AAV/HBV infection. (**A**) 14 days after the fourth immunization, the spleen cells were stimulated with 10 μg/mL HBsAg for 18 hours *in vitro*. Cells were incubated for 6 hours with PMA/Ionomycin (100 ng/mL / 1μg/mL) as a positive control. Th2 was detected by intracellular cytokine. The percentage of IFN-γ^{+}CD4^{+} T cells is presented. (**B**) Representative flow cytometric profiles of IFN-γ^{+}CD8^{+} T cells.(**C**) Representative flow cytometric histograms of HBsAg-specific CTL activity *in vivo* in AAV/HBV infected mice.

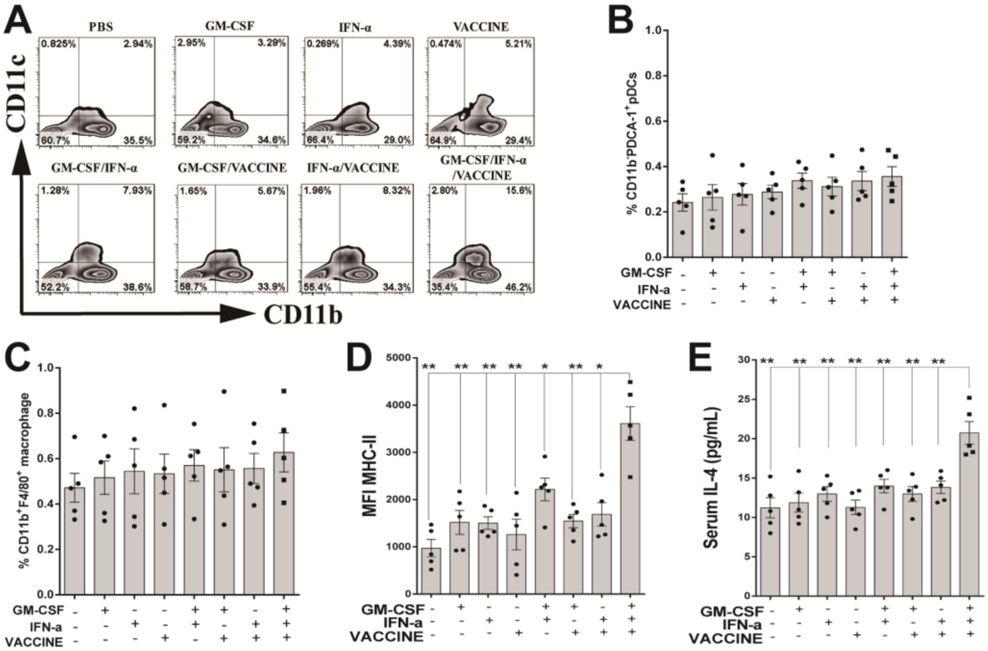

Supplementary Figure 4: GM-CSF/IFN-α/VACCINE promoted both the production and the function of CD11b^{+}CD11c^{+} DC. (**A**) Representative flow cytometric profiles of CD11b^{+}CD11c^{+} DC in blood. (**B–C**) Percentage of CD11b^{+}F4/80^{+} macrophage and CD11b-PDCA^{+} plasmacytoid dendritic cells in blood of AAV/HBV infected mice inoculated with different formulations of the vaccine. (**D**) Comparison of MCH-II on of CD11b^{+}CD11c^{+} DC in blood. (**E**) Serum concentration of IL-4 of AAV/HBV infected mice.

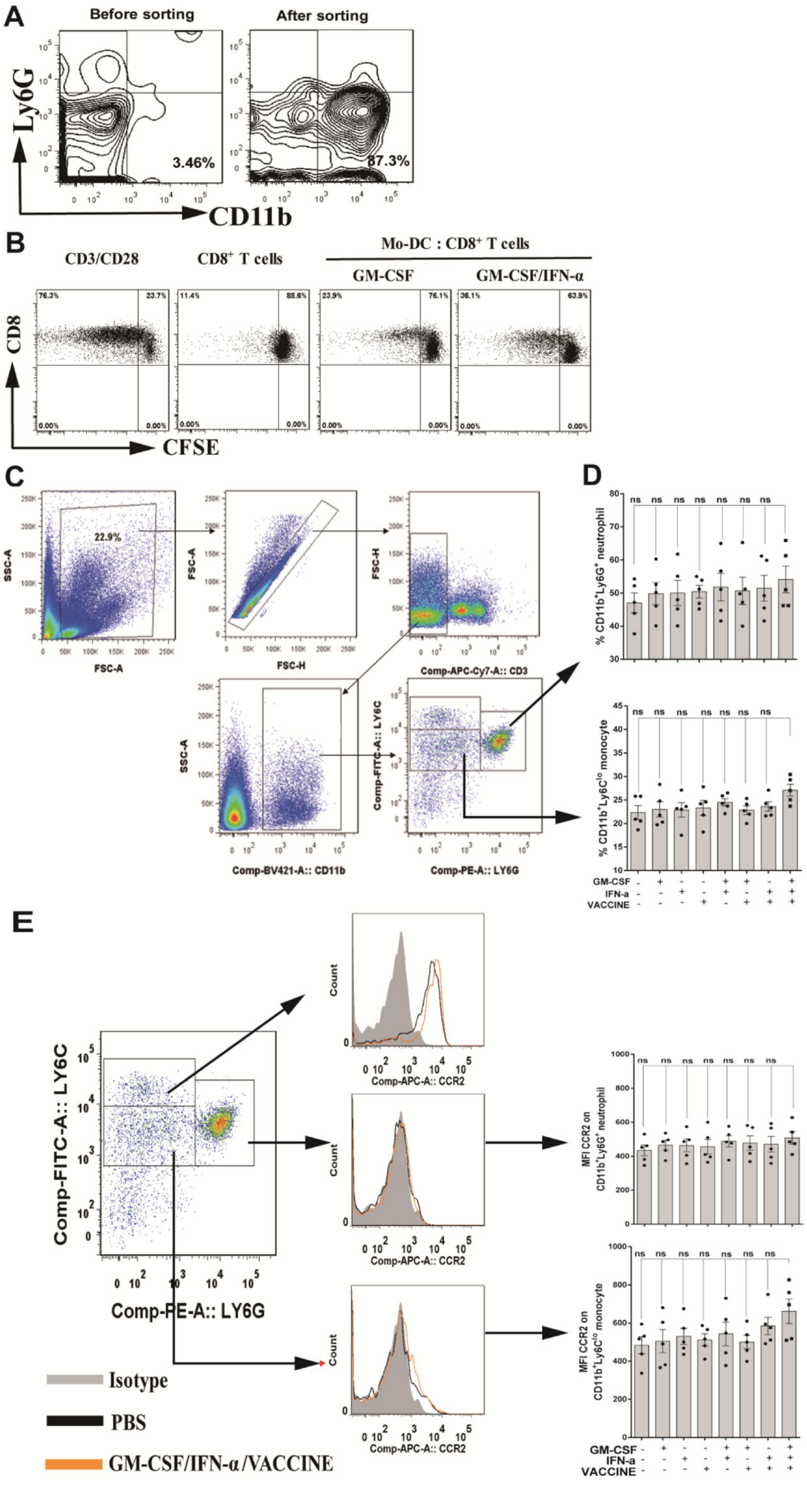

Supplementary Figure 5: GM-CSF plus IFN-α promoted both the production and the function of CD11c$^+$ monocyte derived DC (MoDC) *in vitro* and *in vivo*. (**A**) CD11b$^+$Ly6G$^-$ monocytes were sorted from PBMC of male wild-type C57BL/6 (n = 50). (**B**) Conventional CD8$^+$ T cells were sorted from spleen cells of AAV8-1.3HBV mice and co-cultured with HBsAg (10 μg/mL)-pulsed MoDC which generated from GM-CSF or GM-CSF/IFN-α at ratio 10:1 for 72 hours. CD3/CD28 (1μg/mL / 100 ng/mL) was used as a positive control. (**C**) Flow cytometry gating of inflammatory monocytes (CD3$^-$CD11b$^+$Ly6C^{hi}), circulating monocytes (CD3$^-$CD11b$^+$Ly6C^{lo}), and granulocytes (CD3$^-$CD11b$^+$Ly6G$^+$) in blood of AAV8-1.3HBV-infected mice. (**D**) Percentage of blood Ly6G$^-$Ly6C^{lo} monocytes, and Ly6G$^+$Ly6C$^+$ granulocytes of AAV8-1.3HBV-infected mice treated different formulation vaccine for 3 days. Numbers adjacent to outlined area indicate percent Ly6G$^-$Ly6C^{hi} monocytes (top left), Ly6G$^-$Ly6C^{lo} monocytes (bottom left), and Ly6G$^+$Ly6C$^+$ granulocytes (right). Bars are shown as mean ± SEM. ns, not significan. (**E**) Comparison of MFI CCR2 on Ly6G$^-$Ly6C^{lo} monocytes, and Ly6G$^+$Ly6C$^+$ granulocytes. Data are pooled from two independent experiments with 5 mice per group. Bars are shown as mean ± SEM. ns, not significant.
